# Direct observation of magnetic domain walls in glass-coated submicronic amorphous wires

**DOI:** 10.1038/s41598-024-56359-w

**Published:** 2024-03-08

**Authors:** T.-A. Óvári, G. Ababei, G. Stoian, S. Corodeanu, H. Chiriac, N. Lupu

**Affiliations:** National Institute of Research and Development for Technical Physics, 47 Mangeron Boulevard, 700050 Iași, Romania

**Keywords:** Condensed-matter physics, Materials for devices, Microscopy, Applied physics, Condensed-matter physics

## Abstract

Results on the magnetic domain walls in rapidly solidified magnetostrictive and non-magnetostrictive amorphous submicronic wires are reported. Utilizing Lorentz transmission electron microscopy (LTEM) for the first time in this context, we have visualized and analyzed the domain walls in such ultra-thin amorphous wires. All the investigated samples display vortex magnetic domain walls, regardless of wire composition or diameter. In non-magnetostrictive wires, the domain walls maintain their structure and symmetry under varying magnetic field conditions. In contrast, magnetostrictive wires show an elongation of their domain walls upon magnetic field application, a response linked to the magnetoelastic coupling between magnetostriction and internal stresses induced during wire preparation. This study advances the understanding of magnetization reversal processes in amorphous submicronic wires. The insights gained are crucial for future developments in miniaturized magnetic devices.

## Introduction

Rapidly solidified amorphous glass-coated nanowires and submicronic wires with diameters between 80 and 950 nm have been widely investigated during the last decade^1^. They have been produced for the first time during 2010–2013^2–4^. This was a natural evolution driven by the need for miniaturization from the much better-known amorphous glass-coated microwires, with diameters between 1 and 80 μm^5^, that have been studied more thoroughly during an extensive period (1970s to today)^6,7^.

The preparation of submicronic cylindrical amorphous magnetic wires requires several key adjustments in the actual preparation method^8^, despite using the same general technique as in the preparation of the thicker amorphous microwires, that is the glass-coated melt spinning method^9^. Such ultra-thin amorphous magnetic wires with cylindrical symmetry are usually produced either from highly magnetostrictive alloys, e.g. Fe_77.5_Si_7.5_B_15_ with λ = 25 × 10^–6^, or from nearly zero magnetostrictive ones, e.g. (Co_0.94_Fe_0.06_)_72.5_Si_12.5_B_15_ with λ = – 1 × 10^–7^. FINEMET type alloys, i.e. Fe_73.5_Cu_1_Nb_3_Si_13.5_B_9_, represent another option, due to their ability to vary their magnetostriction with the correct annealing from 25 × 10^–6^ down to 0, due to the structural changes induced by the nanocrystalline phase formation^10^. However, the case of FINEMET submicronic wires is somewhat different, precisely due to these structural changes, and, for the purposes of this work we will only focus on amorphous samples.

Submicronic amorphous wires typically cover diameters between 80 and 950 nm, thus reaching in the nano range for the lower dimensions. The entire range refers to the actual magnetic wires, as the glass coating has thicknesses in the range of μm^11^.

The general characteristic of the magnetic behavior of amorphous glass-coated submicronic wires has been found to be magnetic bistability, i.e. a single step magnetization reversal that occurs when the axially applied magnetic field is larger than a threshold value called switching field, H^*^, and which is also referred to as the large Barkhausen effect when observed in thicker wires, such as the amorphous microwires^12^. Magnetic bistability is mainly emphasized by means of rectangular hysteresis loops^13^.

Although amorphous submicronic wires exhibit magnetic bistability irrespective of composition and magnetostriction^14^, there are some essential differences between highly magnetostrictive and nearly zero magnetostrictive submicronic wires, as well as between the magnetic behavior of nearly zero magnetostrictive submicronic amorphous wires and nearly zero magnetostrictive amorphous microwires. Thus, amorphous submicronic wires with nearly zero magnetostriction are bistable, whilst nearly zero magnetostrictive amorphous microwires typically do not exhibit magnetic bistability, being characterized by an anhysteretic magnetic behavior^15^. On the other hand, both nearly zero and highly magnetostrictive amorphous submicronic wires are magnetically bistable, displaying rectangular hysteresis loops, but there are huge differences between the actual value ranges of their switching fields^16^. Moreover, as the single step axial magnetization reversal entails the propagation of a single magnetic domain wall from one end of the wire towards the other, the velocity and mobility of such a domain wall can differ significantly in submicronic amorphous wires with high magnetostriction in comparison with those having nearly zero magnetostriction. Some of these differences have been ascribed to the differences in magnetostriction, although it was not fully clear whether this alone can account for all the observed discrepancies.

Here, we aim to perform a thorough investigation of such differences, by examining the actual domain walls that can appear in either type of submicronic amorphous wires, highly magnetostrictive or nearly zero magnetostrictive. This is also important for applications since, due to the very large values of the domain wall velocities in such ultra-thin samples, it is key to understand the actual peculiarities of magnetic domain walls in each wire type. The actual investigation of the magnetic domain walls has been performed by means of Lorentz transmission electron microscopy (LTEM), and the results are expected to clarify some of the above-mentioned problems. Furthermore, this is for the very first time that LTEM is used to observe and to visualize magnetic domain walls in submicronic amorphous magnetic wires prepared by means of rapid solidification techniques.

## Results

To substantiate the differences in their magnetic behavior, first we will illustrate the axial hysteresis loops of Fe_77.5_Si_7.5_B_15_ and (Co_0.94_Fe_0.06_)_72.5_Si_12.5_B_15_ amorphous glass-coated submicronic wires with highly positive magnetostriction and nearly zero magnetostriction, respectively.

**Figure 1:**
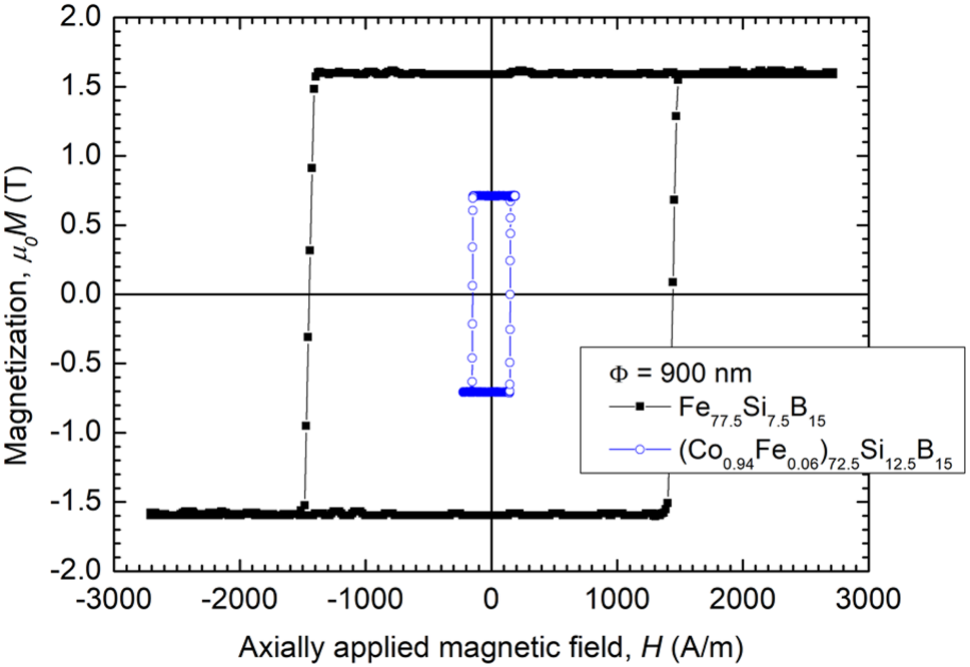
Axial magnetic hysteresis loops in the case of a highly magnetostrictive Fe_77.5_Si_7.5_B_15_ submicronic amorphous wire and a nearly zero magnetostrictive (Co_0.94_Fe_0.06_)_72.5_Si_12.5_B_15_ one, both having Φ = 900 nm in diameter.

Figure 1 shows the axial hysteresis loops of two amorphous submicronic wires—an Fe_77.5_Si_7.5_B_15_ magnetostrictive one, and a (Co_0.94_Fe_0.06_)_72.5_Si_12.5_B_15_ nearly zero magnetostrictive one, respectively, both having 900 nm in diameter.

One can clearly see that, despite sharing similarly shaped hysteresis loops, which indicate magnetic bistability in both cases, the actual values of the magnetic switching field are significantly different. This difference can be even larger in the case of amorphous submicronic wires with diameters below 500 nm^17^.

As we have already mentioned, magnetic bistability implies the propagation of a single magnetic domain wall from one end of a submicronic amorphous wire to the other.

**Figure 2:**
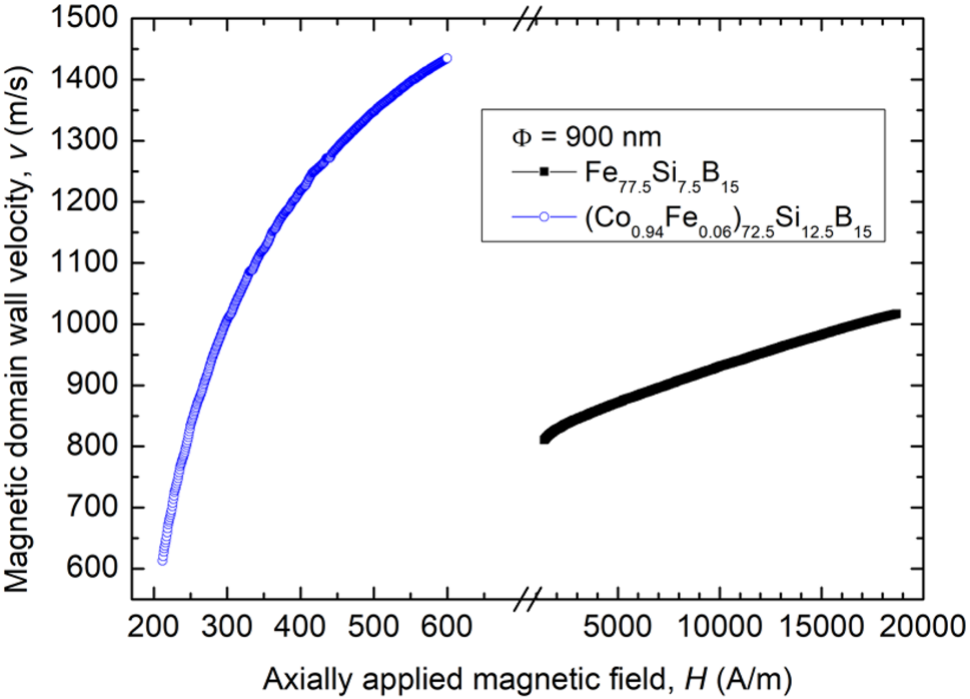
Domain wall velocity vs. axial magnetic field for a highly magnetostrictive Fe_77.5_Si_7.5_B_15_ submicronic amorphous wire and a nearly zero magnetostrictive (Co_0.94_Fe_0.06_)_72.5_Si_12.5_B_15_ one, respectively, both with 900 nm in diameter.

Figure 2 illustrates the velocity of such magnetic domain walls vs. the axially applied magnetic field in the case of two submicronic amorphous samples with 900 nm in diameter: a highly magnetostrictive Fe_77.5_Si_7.5_B_15_ one, and a nearly zero magnetostrictive (Co_0.94_Fe_0.06_)_72.5_Si_12.5_B_15_ one. Again, one observes the totally different field ranges, although the domain wall velocities are not quite as different. The domain wall velocity values can reach much larger values in the case of samples with significantly smaller diameters, such as 4000 m/s for nearly zero magnetostrictive ones with 400 nm in diameter^17^. Domain wall velocity values of 10^3^ m/s and larger are already important for domain-wall-based applications, such as domain wall logic devices and sensors, in which the speed of magnetic logic operations based on domain wall propagation is essential. In order to study more thoroughly and better understand the origin of such large domain wall velocities and mobilities – the latter represented by the slopes of the velocity vs. applied field curves, we have focused on the investigation of the domain wall structures, using for the first time Lorentz transmission electron microscopy to analyze the magnetic domain walls in rapidly solidified amorphous submicronic wires.

Let us study the actual domain walls that appear in such ultra-thin amorphous wires by means of Lorentz microscopy.

**Figure 3:**
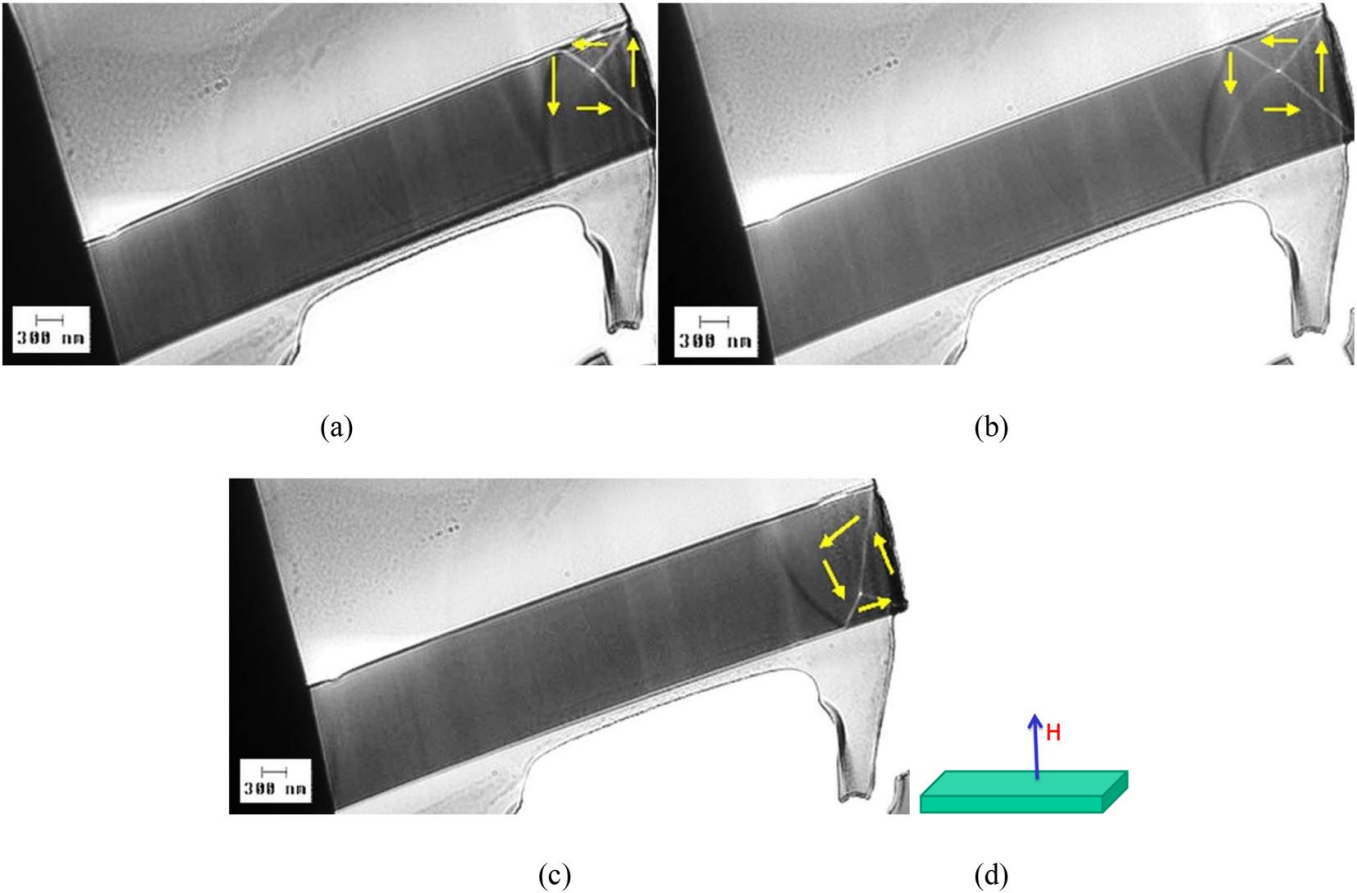
LTEM domain wall images observed on a sample prepared from a nearly zero magnetostrictive (Co_0.94_Fe_0.06_)_72.5_Si_12.5_B_15_ submicronic amorphous wire with 900 nm in diameter, under different values of the applied magnetic field H: (**a**) – 0 A/m; (**b**) – 71.6 kA/m (900 Oe); and (**c**) 87.5 kA/m (1100 Oe). H is applied perpendicular to the plane of the TEM lamella, as shown in the schematic (**d**).

Figure 3 shows magnetic domain wall images observed on a sample prepared from a nearly zero magnetostrictive (Co_0.94_Fe_0.06_)_72.5_Si_12.5_B_15_ submicronic amorphous wire with 900 nm in diameter, under various values of the magnetic field applied perpendicular to the lamella, as indicated on the figure. One can clearly identify a vortex magnetic domain wall towards the right end of the sample in all cases: no field, 71.6 kA/m (900 Oe), and 87.5 kA/m (1100 Oe). This type of LTEM image is typical for a vortex domain wall, with the peak at the intersection of the two lines marking the vortex core^18^.

**Figure 4:**
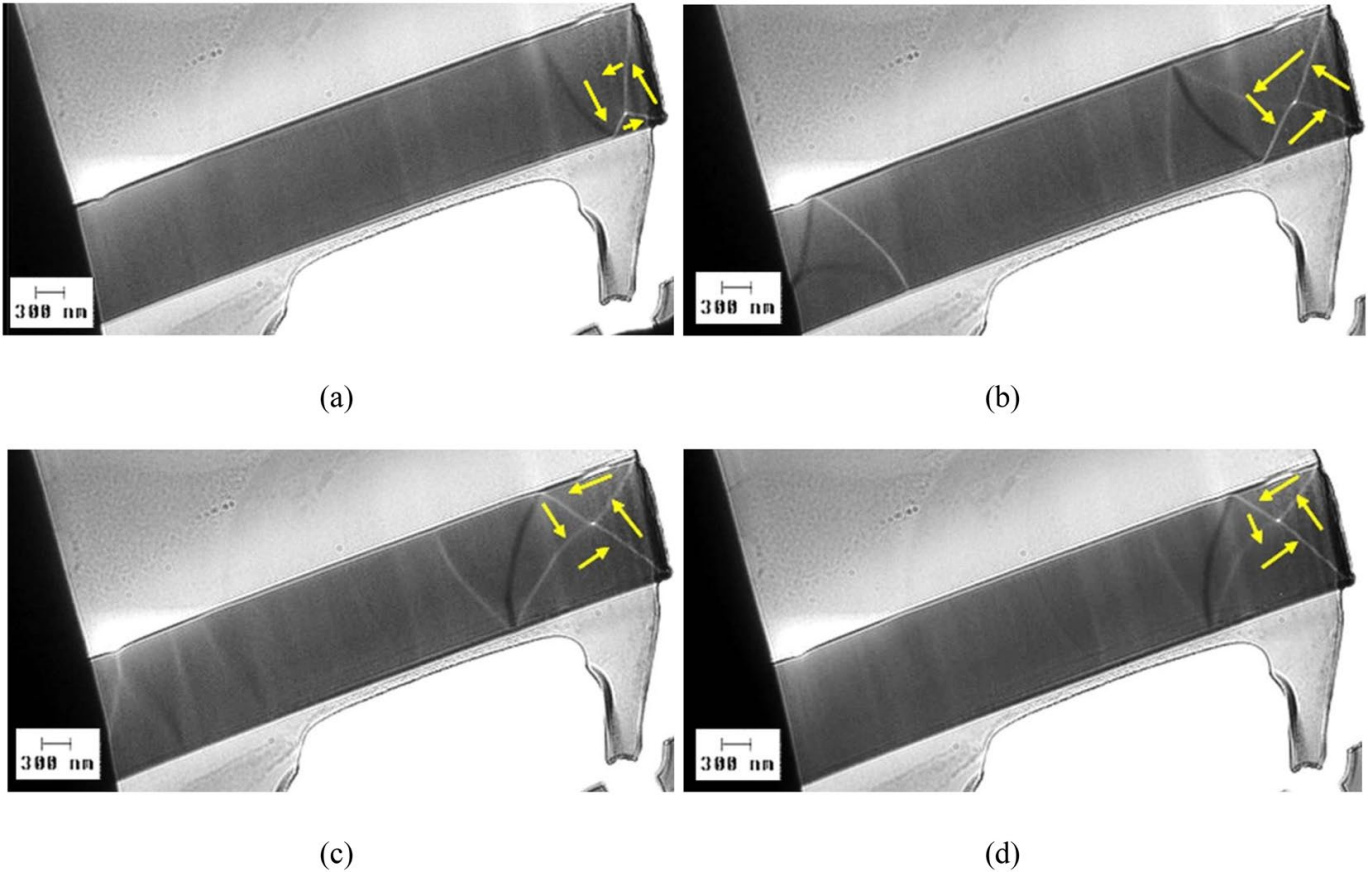
LTEM domain wall images visualized on a sample prepared from a nearly zero magnetostrictive (Co_0.94_Fe_0.06_)_72.5_Si_12.5_B_15_ submicronic amorphous wire with 900 nm in diameter, under an applied magnetic field H = 71.6 kA/m (900 Oe). The lamella is tilted at small angles α from the direction perpendicular to H: (**a**): α = 0° (H is perpendicular to the lamella); (**b**) α = 0.4°; (**c**) α = 1.2°; and (**d**) α = 1.5°.

Tilting the plane of the lamella sample at small angles α with respect to the direction perpendicular to the applied magnetic field H does not alter the vortex structure, although it does result in small displacements of the domain wall, as illustrated in Fig. 4.

**Figure 5:**
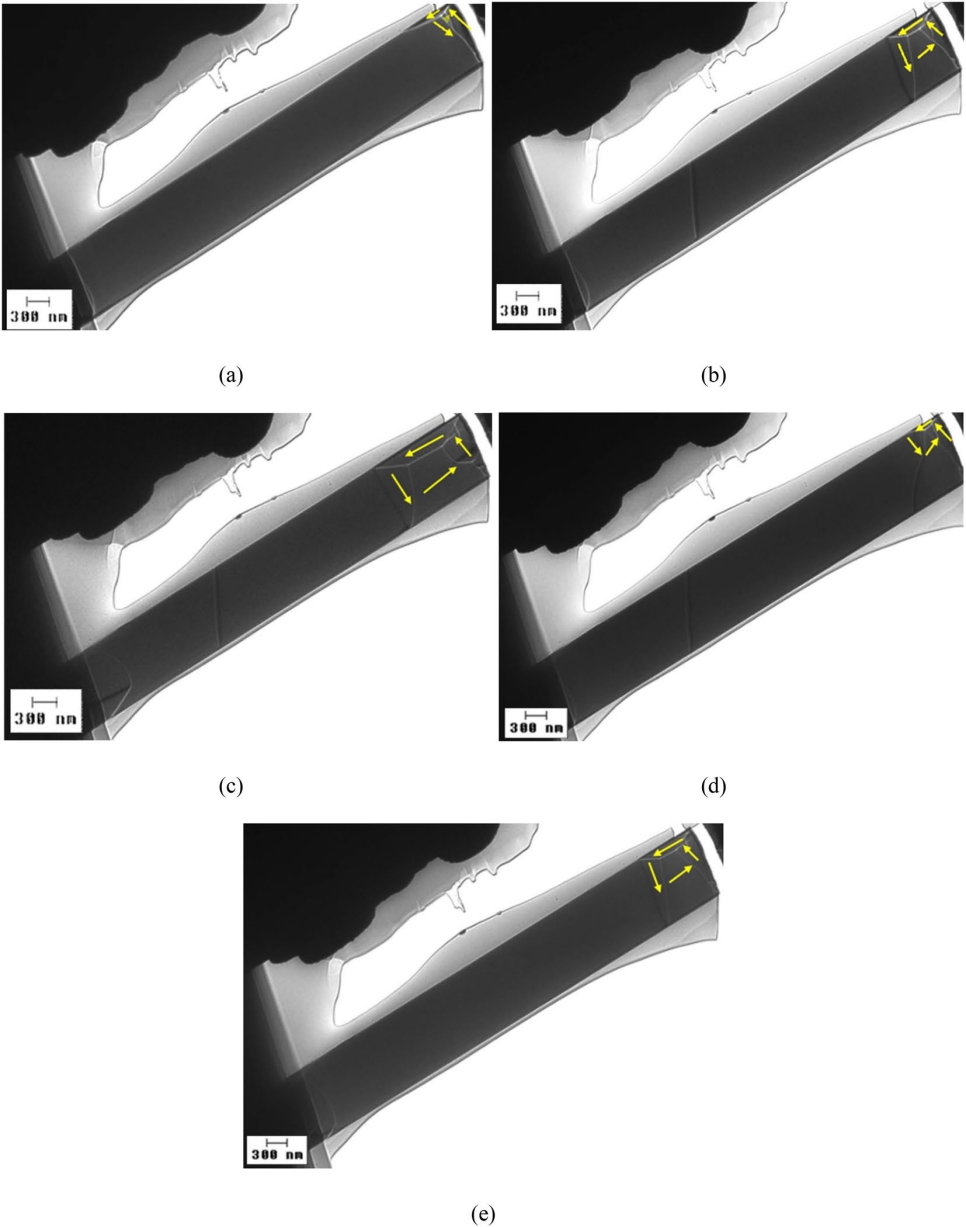
LTEM domain wall images observed on a sample prepared from a highly magnetostrictive Fe_77.5_Si_7.5_B_15_ submicronic amorphous wire with 900 nm in diameter, under various magnetic fields H: (**a**) 0 A/m; (**b**) 23.9 kA/m (300 Oe); (**c**) 59.7 kA/m (750 Oe); and for a constant field of 71.6 kA/m (900 Oe) applied on the lamella tilted at (**d**) 1.5°; and (**e**) 2°, respectively.

In case of an Fe_77.5_Si_7.5_B_15_ amorphous submicronic wire with high magnetostriction, having the same diameter (900 nm), one can see in Fig. 5 that the vortex domain wall displays an elongation that increases with the value of the applied magnetic field. Tilting the lamella sample at small angles has a similar effect as increasing the applied magnetic field.

**Figure 6:**
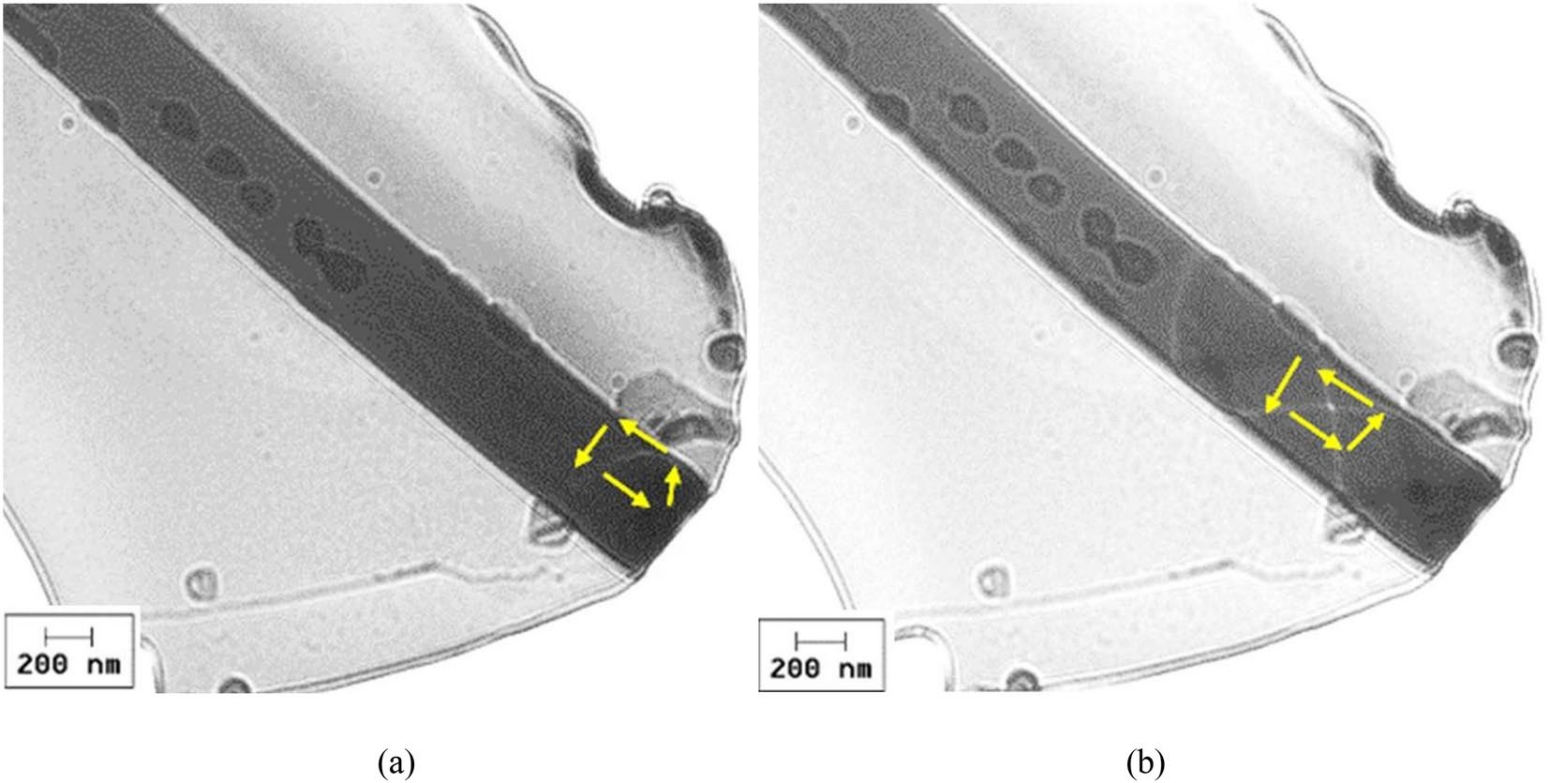
LTEM domain wall images visualized on a sample prepared from a nearly zero magnetostrictive (Co_0.94_Fe_0.06_)_72.5_Si_12.5_B_15_ submicronic amorphous wire with 500 nm in diameter, under an applied magnetic field H of 23.9 kA/m (300 Oe), when the lamella plane is tilted at small angles α with respect to the direction perpendicular to the applied field: (**a**) α = 0.4°; and (**b**) α = 5.5°.

For even thinner wires, i.e. a nearly zero magnetostrictive (Co_0.94_Fe_0.06_)_72.5_Si_12.5_B_15_ amorphous submicronic wire with 500 nm in diameter, LTEM domain wall observations show the same vortex structure of the magnetic domain wall, as illustrated in Fig. 6.

## Discussion

All the investigated magnetic domain walls exhibit vortex structures, irrespective of the sample composition or diameter. This shows that vortex domain walls represent a typical characteristic of rapidly quenched amorphous magnetic submicronic wires with cylindrical symmetry. One can relate this feature to the cylindrical symmetry and to the amorphicity of these wires, namely to the lack of any magnetocrystalline anisotropy. In the (Co_0.94_Fe_0.06_)_72.5_Si_12.5_B_15_ nearly zero magnetostrictive amorphous submicronic wires, the observed symmetry and shape of the magnetic domain walls remain unchanged under various amplitudes and angles of the applied magnetic field, as illustrated by the results presented in Figs. 3 and 4. Moreover, based on the images presented in Fig. 6, a significant variation in diameter does not affect the structure of the magnetic domain wall. The domain walls change their position when subjected to applied field or when the angle under which the field is applied is varied even slightly, but the overall structure and symmetry of the vortex remains the same. Given that such a behavior of the magnetic domain walls in (Co_0.94_Fe_0.06_)_72.5_Si_12.5_B_15_ nearly zero magnetostrictive amorphous submicronic wires has been observed at different sample diameters, one can infer that it is a common characteristic of the magnetic domain walls in low magnetostrictive ultra-thin amorphous wires.

In the case of highly magnetostrictive Fe_77.5_Si_7.5_B_15_ amorphous submicronic wires, the vortex structure of the domain walls is the closest to the symmetry encountered in the domain walls of (Co_0.94_Fe_0.06_)_72.5_Si_12.5_B_15_ amorphous samples only in the absence of an applied magnetic field. As soon as a field is applied, the domain wall exhibits a change in symmetry, an elongation, that increases with the value of the applied field, as shown in Fig. 5. The same behavior was observed when the direction of the applied magnetic field was tilted at small angles. Thus, not only the hysteresis loops and the domain wall velocities exhibit differences in the two types of submicronic amorphous wires, but the actual domain wall structures as well. The main difference is the elongated aspect taken by the vortex domain walls in the Fe_77.5_Si_7.5_B_15_ amorphous submicronic samples with respect to the simple, non-elongated vortices encountered in the (Co_0.94_Fe_0.06_)_72.5_Si_12.5_B_15_ samples.

This difference, alongside the increment of the elongation with the magnetic field, as well as the huge difference in the values of the switching field between the non-magnetostrictive and the highly magnetostrictive amorphous samples, suggest that a typical domain wall in the latter would first elongate when the applied axial magnetic field increases, before depinning and propagating when the applied field reaches the value of the switching field.

One should note that the interpretation of the magnetic field values is challenging, given that the LTEM analysis can be performed solely on lamella-type samples prepared from the actual submicronic amorphous wires, and not on the wire samples as such. Also, the direction of the applied magnetic field acting on the domain walls in such lamella samples is limited to the directions allowed by the LTEM system configuration, and not freely selectable, as in the case of magnetic fields applied on the actual amorphous submicronic wires during magnetization reversal, hysteresis measurements, and domain wall velocity measurements.

Nevertheless, the magnetic domain wall behavior observed by means of LTEM investigations is clearly different between the two types of submicronic amorphous wires: magnetostrictive and nearly zero or non-magnetostrictive. Hence, one can consider that the magnetic domain walls within cylindrical amorphous submicronic wires would behave in a similar manner. Thus, in the magnetostrictive amorphous submicronic wires, the domain wall between the pre-existing end domains with reverse magnetization (created by the demagnetizing field) and the overall magnetization of the wire, will first suffer elongation under the action of the axially applied magnetic field, when this field is below the threshold, below the value of the switching field, and subsequently will suffer depinning and propagate when the axial field reaches the value of the switching field. On the contrary, such behavior is not expected in nearly zero magnetostrictive submicronic amorphous wires, in which no elongation is likely to occur prior to the propagation of the domain wall at magnetization reversal. This different behavior is a consequence of the different magnetostriction constants in the two types of submicronic amorphous wires, as there are no other significant differences between them.

In the highly magnetostrictive amorphous submicronic samples, the magnetoelastic coupling between magnetostriction and the large internal stresses induced during preparation^19^, would lead to a radial easy axis of magnetization in the near-surface region of the wire. Such a distribution of the magnetization, even though located in the near-surface region, would create a stray field that would clearly hinder the free displacement of the pre-existing magnetic domain wall along the wire. This is not the case for the nearly zero magnetostrictive amorphous submicronic wires, in which such an obstructive stray field is not expected, due to the much weaker magnetoelastic coupling.

Consequently, by correlating the hysteresis loops and domain wall velocity data with Lorentz transmission electron microscopy observations of the magnetic domain walls, we have been able to get new insights into the magnetization reversal process in rapidly solidified amorphous submicronic wires. Furthermore, we have got a more comprehensive understanding of the differences between the magnetic behavior of nearly zero magnetostrictive samples and that of highly magnetostrictive ones.

## Methods

Submicronic amorphous glass-coated magnetic wires have been produced using the improved variant of the glass-coated melt spinning method at the National Institute of Research and Development for Technical Physics, Iași, Romania^8^. We have employed Fe_77.5_Si_7.5_B_15_ with positive magnetostriction (λ = 25 × 10^–6^) to prepare highly magnetostrictive samples, and (Co_0.94_Fe_0.06_)_72.5_Si_12.5_B_15_ with λ = – 1 × 10^–7^ to prepare nearly zero magnetostrictive ones, respectively. The diameters of the actual submicronic magnetic amorphous wires have been varied between 500 and 900 nm for the purposes of these investigations.

Direct observation of the magnetic domain walls in the submicronic amorphous samples with both compositions has been performed by means of Lorentz transmission electron microscopy (LTEM), using a Libra 200 MC Carl Zeiss ultra-high-resolution microscope in Fresnel configuration^20^. In order to visualize the domain walls, typical transmission electron microscopy (TEM) lamella preparation has been employed in the case of samples with both compositions. Using a focused ion beam (FIB) system, the glass coating removal has been performed in the initial stage of the process, followed by subsequent thinning of the magnetic wire down to a 100 nm thickness. For an accurate control over the entire processing of the samples, the FIB current has been gradually reduced from ~ 5 nA initially down to 500 pA during the final stages. The typical lamella employed in all the LTEM observations were 50 μm long and 100 nm thick.

**Figure 7:**
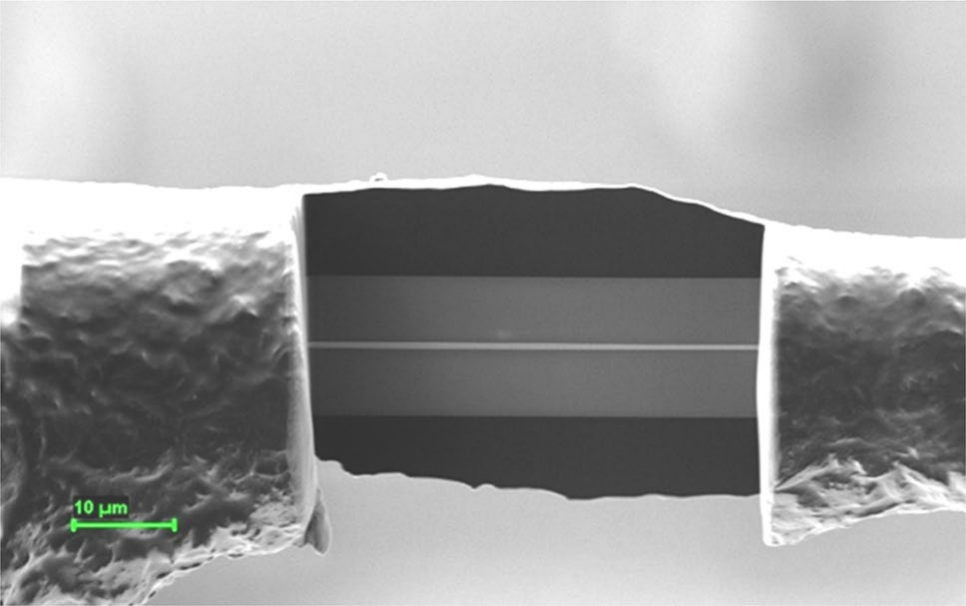
Intermediate stage of a TEM lamella preparation for the LTEM investigations.

Figure 7 shows a scanning electron microscopy (SEM) image taken during a lamella preparation. After the lamella is ready, it is subsequently fixed to the holder and inserted into the TEM, where the magnetic domain walls are observed in Fresnel, or defocus mode^21^. Magnetic domain wall observations have been performed under various values of an applied magnetic field, which has been applied under various angles, as allowed by the LTEM system configuration.

The magnetic field was applied through the objective lens, which is made of two electromagnetic lenses positioned in a Helmholtz geometry. The sample is inserted between these two lenses in a central position. The system allows one to change in fixed steps the dc current through the objective lens, resulting in fixed values of the magnetic field that can be applied to the sample: 0 A/m (0 Oe), 11.9 kA/m (150 Oe); 23.9 kA/m (300 Oe); 35.8 kA/m (450 Oe); 47.75 kA/m (600 Oe); 59.7 kA/m (750 Oe); 71.6 kA/m (900 Oe); 87.5 kA/m (1100 Oe), and 95.5 kA/m (1200 Oe). The magnetic sample can be tilted between 0° and up to ± 15° along its longitudinal axis. For all measurements, the first image of the sample was recorded in focus (classical high resolution transmission electron microscopy image), at 0° tilting angle and in zero magnetic field, this being considered as the reference image. Subsequently, the image is slowly defocused (Fresnel method) until the magnetic domain walls become visible (sample is still at 0° tilting angle and in zero magnetic field). To generate changes in the domain walls, either different value fields are applied through the objective lens at a fixed angle of the sample holder, or the magnetic field is kept constant, and the sample is tilted at various angles. LTEM images have been recorded at the minimum values of the angles at which, for a given value of the applied field, modifications of the observed domain wall have been observed.

In this way, one can study the effect of various magnetic fields on the magnetic domain walls. Lorentz microscopy data has been correlated with hysteresis loop measurements and domain wall velocity measurements for a comprehensive investigation. Hysteresis loops have been measured using an inductive technique developed specifically for these ultra-thin submicronic amorphous samples^22^, while the velocity of the magnetic domain walls has been measured using an improved Sixtus-Tonks-based method, also dedicated to such submicronic cylindrical amorphous wires^23^.

## Conclusions

This study presents an investigation of the magnetic domain walls in rapidly solidified amorphous submicronic wires, focusing on wires with both high and nearly zero magnetostriction. Employing Lorentz transmission electron microscopy (LTEM), our research marks the first time this technique has been used to visualize magnetic domain walls in such ultra-thin amorphous wires.

Our findings reveal that irrespective of the wire composition or diameter, all investigated magnetic domain walls exhibited vortex structures, a characteristic attributed to the wires' cylindrical symmetry and lack of magnetocrystalline anisotropy. Notably, in nearly zero magnetostrictive amorphous submicronic wires, the structure and symmetry of the magnetic domain walls remained constant under varying magnetic field amplitudes and angles.

In contrast, highly magnetostrictive amorphous submicronic wires demonstrated a notable difference in domain wall behavior. The vortex structure of these domain walls elongates significantly with the application of a magnetic field, indicating a distinct response compared to nearly zero magnetostrictive samples. This behavior can be linked to the magnetoelastic coupling and the substantial internal stresses induced during wire preparation.

By correlating hysteresis loops and domain wall velocity data with LTEM observations, we have gained novel insights into the magnetization reversal process in these wires, advancing the understanding of their overall magnetic behavior. Potential applications in advanced miniaturized magnetic devices, where understanding magnetic domain wall dynamics is crucial, could greatly benefit from these results. For instance, domain wall velocity values of 10^3^ m/s and larger are important for domain-wall-based applications, such as domain wall logic devices and microsensors, in which the speed of magnetic logic operations or response to external stimuli using the fast domain wall propagation is essential.

## Data Availability

Data underlying the results presented in this paper are available from the corresponding author (nicole@phys-iasi.ro) upon reasonable request.
